# Relationship between indoor noise perception and remote work during the COVID-19 pandemic

**DOI:** 10.1371/journal.pone.0286481

**Published:** 2023-06-09

**Authors:** Sang Hee Park, Hye-Kyung Shin, Kyoung-Woo Kim

**Affiliations:** Department of Building Research, Korea Institute of Civil Engineering and Building Technology, Goyang-si, Gyeonggi-do, South Korea; Conestoga College Institute for Technology and Advanced Learning, CANADA

## Abstract

The coronavirus disease 2019 (COVID-19) pandemic has had a major influence on working patterns worldwide, given the various lockdown periods and the shift to remote working. As people’s noise perception is known to be closely linked with their work performance and job satisfaction, investigating the noise perception in indoor spaces, especially in situations where people work from home, is crucial; however, studies on this aspect are limited. Thus, here, this study aimed to investigate the relationship between indoor noise perception and remote work during the pandemic. The study assessed how people who worked from home perceived indoor noise, and how it related with their work performance and job satisfaction. A social survey was conducted with respondents who worked from home during the pandemic in South Korea. A total of 1,093 valid responses were used for data analysis. Structural equation modeling was used as a multivariate data analysis method to simultaneously estimate multiple and interrelated relationships. The results showed that indoor noise disturbance significantly affected annoyance and work performance. Annoyance with indoor noise affected job satisfaction. Job satisfaction was found to have a significant impact on work performance, particularly on two dimensions of the work performance that are crucial for achieving organizations’ goals. Moreover, one dimension of the work performance had a significant impact on annoyance. The study proposed that reducing negative perception of indoor noise and improvement of job satisfaction can lead to the maximization of one’s work performance when working from home.

## 1 Introduction

The coronavirus disease 2019 (COVID-19) has spread widely since December 2019. Since its outbreak, the number of infections and deaths has increased worldwide. As of March 30th, 2023, the World Health Organization (WHO) dashboard has reported 30,773,460 confirmed cases and 34,223 deaths due to COVID-19 in South Korea. Notably, in early 2022, South Korea experienced a surge in confirmed cases, with a record high of 621,127 cases reported on March 17th of that year. According to the Korean Ministry of Employment and Labor, the number of remote workers in 2021 was 1,140,000, an increase of over 12 times from 95,000 in 2019 before the COVID-19 outbreak [1]. Moreover, a survey conducted by the Korea Enterprises Federation [2] reported that a considerable number of businesses in South Korea have adopted remote work in response to the pandemic. Specifically, the survey revealed that approximately 72.7% of the top 100 industries in the country implemented remote work arrangements in 2022. The pandemic has had a huge impact on many aspects of our society, including population, politics, economy, environment, culture, and individuals’ lifestyles. In particular, there was a huge change in people’s life style, wherein they have been spending more time indoors. The latest American Time Use Survey shows how people’s usage of time has been affected by the pandemic [3]. It was found that, in 2020, individuals stayed alone for 57 minutes longer a day than they did in 2019. Moreover, individuals spent 1 hour and 33 minutes less time with people outside their household, while they spent 31 minutes longer with household members only.

Before the COVID-19 pandemic, a significant proportion of people used to spend most of their time indoors. Research has indicated that more than 90% of individuals reported they spend the majority of their time inside buildings such as homes, offices, and schools [4]. However, with the implementation of social distancing policies during the pandemic, the amount of time spent indoors has significantly increased. In fact, recent research suggests that some individuals may remain indoors 100% of the time due to the pandemic [5]. This prolonged indoor stay has resulted in increased exposure to various types of outdoor and indoor noises, making the acoustic environment a critical factor affecting occupants’ well-being and health [6]. Studies have reported a surge in noise complaints during pandemics. A recent analysis of noise complaint data collected over ten years in New York City revealed an increased noise complaints during the pandemic, especially among low-income residents [7]. In addition, a case study conducted in Greater London found that the number of noise complaints increased by 48% after the COVID-19 outbreak, with construction activities and neighborhood noise being the primary sources of complaints [8]. These findings suggest that the pandemic has exacerbated noise complaints regarding outdoor noise.

Indoor noise, namely, noise generated by people who share the same indoor space, has become one of the major noise sources. During the pandemic, in particular, there has been increased focus on indoor noise within homes, highlighting the importance of considering the acoustic environment not only in public spaces, but also in private residences. In Turkey, a study was conducted to assess changes in public perception of the acoustic environment during the pandemic [9]. The study compared respondents’ noise annoyance levels from one year prior to the pandemic to those during the pandemic. The results showed a significant decrease in annoyance with traffic noise, while annoyance with indoor noise in dwellings significantly increased. A study conducted in India analyzed noise levels for two months during the lockdown [10]. The study found a significant decrease in noise levels during the lockdown period. However, when the lockdown was eased, there was an increase in noise levels, likely due to the resumption of traffic and industrial activities. This seems to be a result that contradicts the previous study [8] that introduced an increase in complaints due to outdoor noises such as due to construction noise neighborhood noise. However, despite the decrease in noise levels, it could be evidence that noise complaints may increase as residents spend more time indoors. Evidence for this can be found in another study conducted in India [11]. The study reported that residents perceived the indoor environment to be noisier during the lockdown, despite the significant reduction in outdoor noise [11]. The study also examined the effects of indoor soundscape on residents’ productivity and online learning, and found that indoor soundscape had adverse impacts. There is also a study that reported somewhat different results. A social survey conducted in Argentina during the lockdown found that residents perceived less mechanical sounds and more natural sounds, which were associated with feelings of tranquility and happiness [12]. Another cross-sectional study compared survey responses collected from Italy and several other countries. The study found most survey respondents reported that the outdoor environment had become quieter compared to before the pandemic outbreak [13]. However, half of the participants in Italy and most of those in the international survey responded that they perceived the indoor environment to be noisier after the outbreak. The studies introduced above have demonstrated the significant impact of the pandemic on the acoustic environment, with changes in noise levels, type of noise sources, and residents’ noise perception. While variations in results have been reported, the studies have suggested that the pandemic has altered the acoustic environment in a notable way.

If an individual’s activity is disturbed by noise, he/she is likely to perceive noise as annoyance. Disturbance refers to the degree to which indoor noise disrupts an individual’s activities. Annoyance, on the other hand, refers an individual’s level of irritation towards specific noise sources. These two variables are frequently measured separately in the literature [14, 15], and research on different types of noise sources has revealed the link between disturbance and annoyance. A study that assessed large public health survey data collected in southern Sweden reported that exposure to road traffic noise led to disturbance in conducting daily activities, noise annoyance, and general health problems [16]. Particularly, the study reported that road traffic noise higher than 55 dB (L_Aeq,24hr_) disturbed the residents’ rest and sleep by 28.1% and 26.6%, respectively. Another study that performed a survey in Canada found that road traffic noise disturbed the daily activities of residents, including sleeping, conversations between people, watching TV or listening to radio, and activities such as reading and writing [17]. These disturbances were found to be related to annoyance due to high noise levels. A social survey study carried out in a region near an airport in the Netherlands examined how disturbances affected annoyance [18]. The study reported that aircraft noise disturbed the residents’ sleep and rest, conversation, and activities that need concentration. Also, disturbance had a significant impact on noise annoyance. The close relationship between disturbance and annoyance has also been acknowledged in studies on neighbor noise [19, 20]. Neighbor noise has been found to significantly disturb various activities, including sleep, rest, conversation, reading and other quiet activities, resulting in noise annoyance among affected individuals [19]. However, it remains unknown whether this also applies to indoor noise exposure.

### 1.1 The influence of COVID-19 on work

The pandemic has influenced working patterns worldwide. The Organization for Economic Co-operation and Development (OECD) reported that 47% of employees in Australia, France, and the United Kingdom worked remotely during lockdown periods in 2020 [21]. Advancements in technology have helped many countries shift their work patterns to remote working relatively easily and promptly. According to a report on remote work, jobs related to education, training, and libraries can manage 98% of the work from home [22]. Also, it reported that even the entire percentage of computer and mathematical jobs can be performed from home. Although there may be differences depending on job characteristics, many researchers have recognized the possibility of remote work [23]. The demonstrated feasibility of remote work by many companies may accelerate technological advancements [24], paving the way for swift transitions to remote work in similar or necessary situations in the future. Regardless of how much technology has improved and how far industries have found it efficient, it is still unknown how individual employees perceive working remotely, particularly during the period when they are exposed to indoor noise.

Whether remote work is implemented or not, some studies have examined the impact of the pandemic on employees’ work performance. A cross-sectional study performed in Japan assessed full-time employees’ mental health and work performance [25]. It was found that the manner in which the workplace responded to COVID-19 (e.g., preventive measures to reduce the risk of infection) correlated with employees’ fear and worry related to COVID-19, psychological distress, and work performance. Another study examined how Spanish professionals’ emotional intelligence influenced the link between COVID-19-generated stress and work performance [26]. The study found that emotional intelligence was crucial for reducing stress and securing work performance. In addition, a study examined the relationships among COVID-19-induced work stressors, job performance, distress, and life satisfaction in a sample of professionals in India, and found that individuals’ life satisfaction can be lowered due to reduced work performance [27].

### 1.2 Acoustic environment of workplace

Acoustic environment has been found to have a significant impact on employees’ work performance, as shown by a longitudinal study that assessed occupant perceptions who had transitioned from private offices to open-plan offices [28]. The study revealed that the acoustic environment in open-plan offices resulted in increased distraction, difficulty in concentration, frequent use of coping strategies, and decreased speech privacy. According to a comprehensive review on indoor environmental quality in office settings, the acoustic environment of an office is strongly linked to its design [29]. This review highlighted the challenge of speech privacy in cubicle or cellular office layouts, and suggested that careful selection of materials for partitions and an appropriate office layout can be effective strategies for enhancing acoustic comfort. However, not many studies have investigated the impacts of acoustic environments in remote workplace such as houses.

The pandemic has led to a significant increase in remote work, resulting in a growing interest in the indoor environmental quality of remote workplaces. A recent study in Italy developed a tool to measure the impact of remote work environments on workers’ well-being, including 15 items categorized into five indicators, such as acoustic comfort [30]. A case study conducted in Canada examined how noise conditions affected people’s ability to work from home. The study reported that about 25% of the respondents found that noise conditions had a negative impact on their work [31]. Moreover, the study assessed how each of the noise sources affected different activities, and found indoor noise made by other occupants in the same house was the most disturbing. Another social survey was conducted in Italy, assessing how employees perceived noise annoyance in the remote working environment [32]. The study reported that the major source of disturbance was noise of people (e.g., talking). More recent research has explored how people who worked from home in London perceived indoor soundscapes during the pandemic, which found that more people at home contributed to the perception of the indoor acoustic environment as intrusive and uncontrollable when working from home [33]. However, there is still a need for case studies in other cultures and countries to obtain a more diverse understanding of how noise conditions affect remote work. Also, the use of validated questionnaires for assessing work performance may be necessary to improve the accuracy of the results obtained from different studies. Furthermore, in most previous studies, reports have been made on the effect of noise perception on work performance, which provides insights into ways to improve work performance by reducing negative noise perception. However, traditional stress theory proposes a transactional relationship between the appraisal, coping, and consequence of stress [34]. This theoretical model encompasses the concept of after-effects that influence the perception of stress from subsequent exposure to stressors resulting from the appraisal and coping behaviors associated with stress. Experiences of stress do not occur through a one-way direct effect between two variables. Multiple variables interact, and the series of experiences of exposure to stressors influence subsequent experiences of exposure to stressors. There have been no cases applying this theoretical model to the relationship between noise annoyance and work performance. Therefore, this study considers work performance as a kind of consequence and examines how it affects noise annoyance as a stressor in the appraisal process.

Various factors have been known to influence an individual’s work performance. Work commitment is one such factor that has been shown to impact job satisfaction, performance, turnover intentions, and turnover [35]. While work engagement is also important for enhancing performance, it alone cannot be considered a determinant [36]. Additionally, the relationship between work performance and job satisfaction is widely recognized [37]. When employees are satisfied with their job, they tend to perform better, resulting in higher productivity. Additionally, satisfied employees are less likely to quit their jobs, reducing turnover rates [38]. Several case studies conducted in different countries have demonstrated a significant link between employees’ job satisfaction and their work performance [39–41]. However, most of these studies were based on data collected from employees working in office environments, and little is known about the relationship between job satisfaction and work performance in the context of remote work. Thus, it is important to examine how job satisfaction and work performance are related in this new work environment.

### 1.3 Hypotheses

Drawing on the existing literature and identifying gaps for further investigation, this study posits the following hypotheses, primarily examining the relationship between noise perception variables, namely disturbance and annoyance, and remote work experience variables, specifically work performance and job satisfaction, during the COVID-19 pandemic. The proposed hypotheses are denoted as follows (H):

*H1*: *Disturbance–Annoyance*
  - *Disturbance caused by indoor noise increases indoor noise annoyance*.
*H2*: *Disturbance–Work performance*
  - *H2a*: *Disturbance caused by indoor noise reduces task performance*.
  - *H2b*: *Disturbance caused by indoor noise reduces contextual performance*.
  - *H2c*: *Disturbance caused by indoor noise increases counterproductive work behavior*.
*H3*: *Work performance–Annoyance*
  - *H3a*: *Task performance reduces affects indoor noise annoyance*.
  - *H3b*: *Contextual performance reduces indoor noise annoyance*.
  - *H3c*: *Counterproductive work behavior increases indoor noise annoyance*.
*H4*: *Annoyance–Job satisfaction*
  - *Indoor noise annoyance reduces job satisfaction*.
*H5*: *Job satisfaction–Work performance*
  - *H5a*: *Job satisfaction increases task performance*.
  - *H5b*: *Job satisfaction increases contextual performance*.
  - *H5c*: *Job satisfaction reduces counterproductive work behavior*.

Based on the hypotheses above, mediation effects are hypothesized as follow (MH):

*MH1*: *Indoor noise annoyance is a mediating variable between disturbance and job satisfaction*.
*MH2*: *Indoor noise annoyance is a mediating variable between work performance and job satisfaction*.
*MH3*: *Work performance is a mediating variable between disturbance and annoyance*.
*MH4*: *Work performance is a mediating variable between job satisfaction and indoor noise annoyance*.
*MH5*: *Job satisfaction is a mediating variable between indoor noise annoyance and work performance*.

## 2 Methods

### 2.1 Hypothesized model

**Fig 1:**
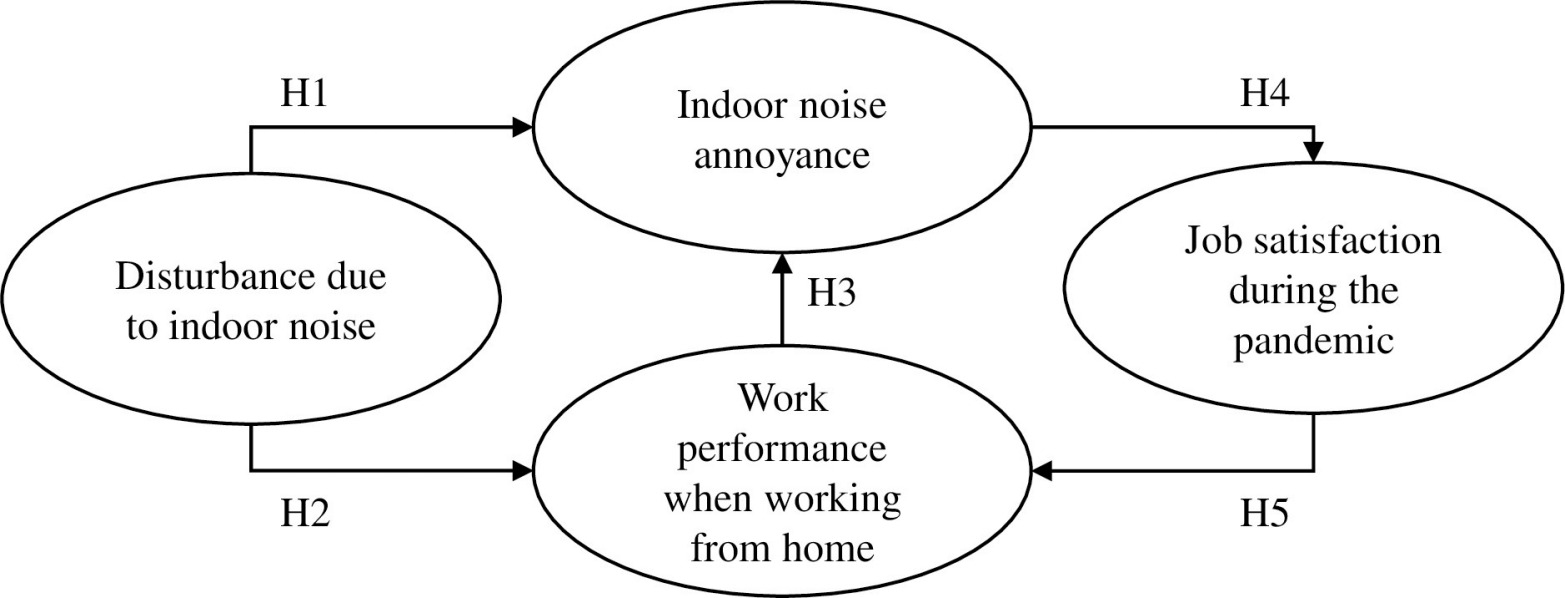
Hypothesized path model.

Fig 1 presents the hypotheses of this model. The model contains hypothesized paths between noise disturbance, noise annoyance, work performance, and job satisfaction. Work performance was measured in three dimensions of task performance, contextual performance, and counterproductive work behavior.

### 2.2 Participants

The online questionnaire survey was conducted between September and October in 2022. The recruitment of the respondents was done using a link which was posted online and sent out via emails. Only those who were residents living in multi-family housing buildings in South Korea, aged 19 years or older, and had worked from home due to the COVID-19 pandemic were screened to participate in the survey. To carry out structural equation modeling (SEM), the study aimed to collect more than the recommended sample size of 300 for this analysis method [42]. In total, 1,105 responses were collected. After excluding 12 outliers, 1,093 responses were used for the data analysis.

This study analyzed valid data collected from 1,093 respondents. Responses were obtained from 545 males and 548 females. The range of age of the participants was 21~74 years (mean = 42.3, SD = 9.9). The percentage of participants living in houses with floor areas between 59 and 84 m^2^ was 38.3%, while those living in houses with floor areas between 84 and 116 m^2^ accounted for 30.5% of the total population. More than half (67.4%) of the participants reported that they had three rooms in their houses. Participants living with two and three other people accounted for 30.3% and 34.1%, respectively. Among the total number of participants, 62.5% owned houses. Table 1 summarizes the details of the participants.

**Table 1:** Demographic characteristics of the participants (*N* = 1,093).

	*n*	%
**Age**		
20s	108	9.9
30s	337	30.8
40s	358	32.8
50s	239	21.9
60s	50	4.6
70s	1	0.1
**Sex**		
Male	545	49.9
Female	548	50.1
**Floor area (m** ^**2**^ **)**		
Smaller than 59	263	24.1
59 to 84	419	38.3
84 to 116	333	30.5
Larger than 116	78	7.1
**Number of people living in the house**		
1	127	11.6
2	196	17.9
3	331	30.3
4	373	34.1
5 or more	66	6.0
**Number of rooms in the house**		
1	47	4.3
2	192	17.6
3	737	67.4
4 or more	117	10.7
**House ownership**		
Owned	683	62.5
Rented (annual)	296	27.1
Rented (monthly)	101	9.2
Other	13	1.2

### 2.3 Questionnaire

Table 2 lists the survey question items used in this study. Firstly, the survey utilized an existing self-report scale to evaluate work performance, which was assessed in three distinct dimensions [43]. It contained five items on task performance (PF_A), eight items on contextual performance (PF_B), and five items on counterproductive work behavior (PF_C). Each of the questions was asked with the phrase “While I was working from home due to the COVID-19 outbreak…” to assess the perception during the pandemic. Secondly, the survey contained four question items to measure how residents’ activities were disturbed by indoor noise during the pandemic. Drawing on prior research on noise disturbance, the survey items related to disturbance (DIS) assessed the impact of indoor noise on different activities [19]: disturbance with work, concentration, rest/sleep, and leisure/conversation. Thirdly, the survey included questions on various sources of indoor noise that led to annoyance (AN) and were developed based on existing literature [19]. The questions measured annoyance due to different sources of noise such as footsteps, voice, noise due to working/studying from other people in the house, and noise from TV, music, games, or radio from within the house. Finally, job satisfaction (JSAT) during the pandemic was assessed using a single question. All questions were presented on a 7-point (0 “Do not agree at all” ~ 6 “Completely agree”) scale [44]. Written informed consent was obtained from all respondents. Ethical approval was obtained from the Public Institutional Review Board designated by the Ministry of Health and Welfare of South Korea (approval no. P01-202204-01-020).

**Table 2:** Question items used in the survey.

Latent variable		Observed variable	Question item
Work performance (PF)			While I was working from home due to the COVID-19 outbreak…
	Task performance (PF_A)	PF_A1	. . .I managed to plan my work so that I finished it on time
		PF_A2	. . .I kept in mind the work result I needed to achieve
		PF_A3	. . .I was able to set priorities
		PF_A4	. . .I was able to carry out my work efficiently
		PF_A5	. . .I managed my time well
	Contextual performance (PF_B)	PF_B1	. . .On my own initiative, I started new task when my old tasks were completed
		PF_B2	. . .I took on challenging tasks when they were available
		PF_B3	. . .I worked on keeping my job-related knowledge up-to-date
		PF_B4	. . .I worked on keeping my work skills up-to-date
		PF_B5	. . .I came up with creative solutions for new problems
		PF_B6	. . .I took on extra responsibilities
		PF_B7	. . .I continually sought new challenges in my work
		PF_B8	. . .I actively participated in meetings and/or consultations
	Counterproductive work behavior (PF_C)	PF_C1	. . .I complained about minor work-related issues at work
		PF_C2	. . .I made problems at work bigger than they were
		PF_C3	. . .I focused on the negative aspects of situation at work instead of the positive aspects
		PF_C4	. . .I talked to colleagues about the negative aspects of my work
		PF_C5	. . .I talked to people outside the organization about the negative aspects of my work
Disturbance by indoor noise (DIS)		DIS1	Indoor noise at my house disturbed me when I worked from home
		DIS2	Indoor noise at my house disturbed me when I was concentrating (e.g., studying, reading etc.)
		DIS3	Indoor noise at my house disturbed my rest or sleep
		DIS4	Indoor noise at my house disturbed my leisure or conversation
Annoyance with noise from home (AN)		AN1	How much were you annoyed by the footstep noise of someone in your house?
		AN2	How much were you annoyed by the voice of someone in your house?
		AN3	How much were you annoyed by the sounds of remote working/studying of someone in your house?
		AN4	How much were you annoyed by the sounds of TV, music, games, and radio played in your house?
Job satisfaction (JSAT)		JSAT	How satisfied are you with your work since the COVID-19 outbreak?

### 2.4 Statistical analysis

The hypothesized model was tested using SEM to estimate multiple and interrelated relationships simultaneously, calculate the measurement error in the estimation process, and describe a model that explains the entire set of relationships [45]. Data were analyzed using IBM SPSS and AMOS for Windows, version 26.0.

## 3 Results

**Fig 2:**
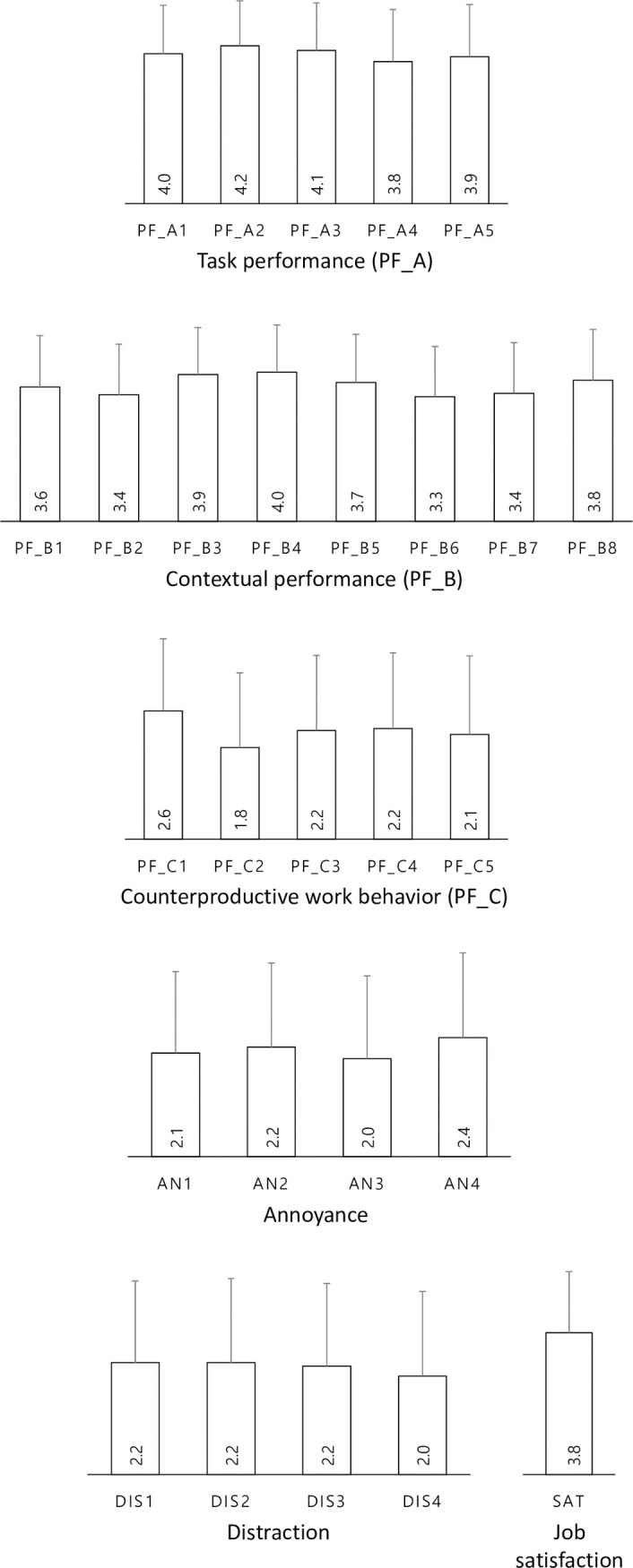
Mean values of each question’s responses (error bars indicate standard deviation).

Fig 2 presents mean values of each question’s responses. It was found that the five subscales of task performance (PF_A) and eight subscales of contextual performance (PF_B) were higher than those of counterproductive work behavior (PF_C). In annoyance ratings, annoyance with sounds of TV, music, games, and radio (AN4) was the highest. For the responses on disturbance, that of leisure or conversation was found to be the lowest (DIS4).

The present study conducted Confirmatory Factor Analysis (CFA) to assess the reliability and validity of the proposed model. To evaluate the internal consistency reliability of each latent variable, Cronbach’s alpha and Composite Reliability (CR) were utilized, where values greater than 0.7 were deemed satisfactory [46, 47]. Furthermore, the convergent validity of the model was assessed by calculating the Average Variance Extracted (AVE). An AVE value of 0.5 or higher indicates that the construct accounts for more than half of the variance of its indicators [48]. As shown in Table 3, the results demonstrated that all values exceeded the recommended cut-off level, and the factor loadings of the observed variables were statistically significant (p < .001).

**Table 3:** Reliability and validity of the model.

Latent variable		AVE	CR	α
Work performance	Task performance (PF_A)	0.714	0.926	0.913
	Contextual performance (PF_B)	0.643	0.935	0.933
	Counterproductive work behavior (PF-C)	0.677	0.913	0.901
Disturbance by noise from home (DIS)		0.773	0.932	0.950
Annoyance with noise from home (AN)		0.747	0.922	0.923

To examine discriminant validity, the Fornell-Larcker criterion was employed [48, 49]. As shown in Table 4, the diagonal values represent the square root of each construct’s AVE, while the nondiagonal values indicate the correlations between the latent variables. According to the criterion, the square root of each construct’s AVE should be greater than its correlation with other constructs. Consistent with this criterion, the results indicate that the square root of each construct’s AVE was larger than its correlation with other constructs, suggesting that discriminant validity was established.

**Table 4:** Result of the Fornell-Larcker criterion analysis.

Latent variables	DIS	AN	PF_A	PF_B	PF_C
Disturbance by noise from home (DIS)	**0.879**				
Annoyance with noise from home (AN)	0.862	**0.864**			
Task performance (PF_A)	-0.208	-0.187	**0.845**		
Contextual performance (PF_B)	-0.131	-0.103	0.825	**0.802**	
Counterproductive work behavior (PF-C)	0.363	0.386	-0.105	-0.028	**0.823**

**Fig 3:**
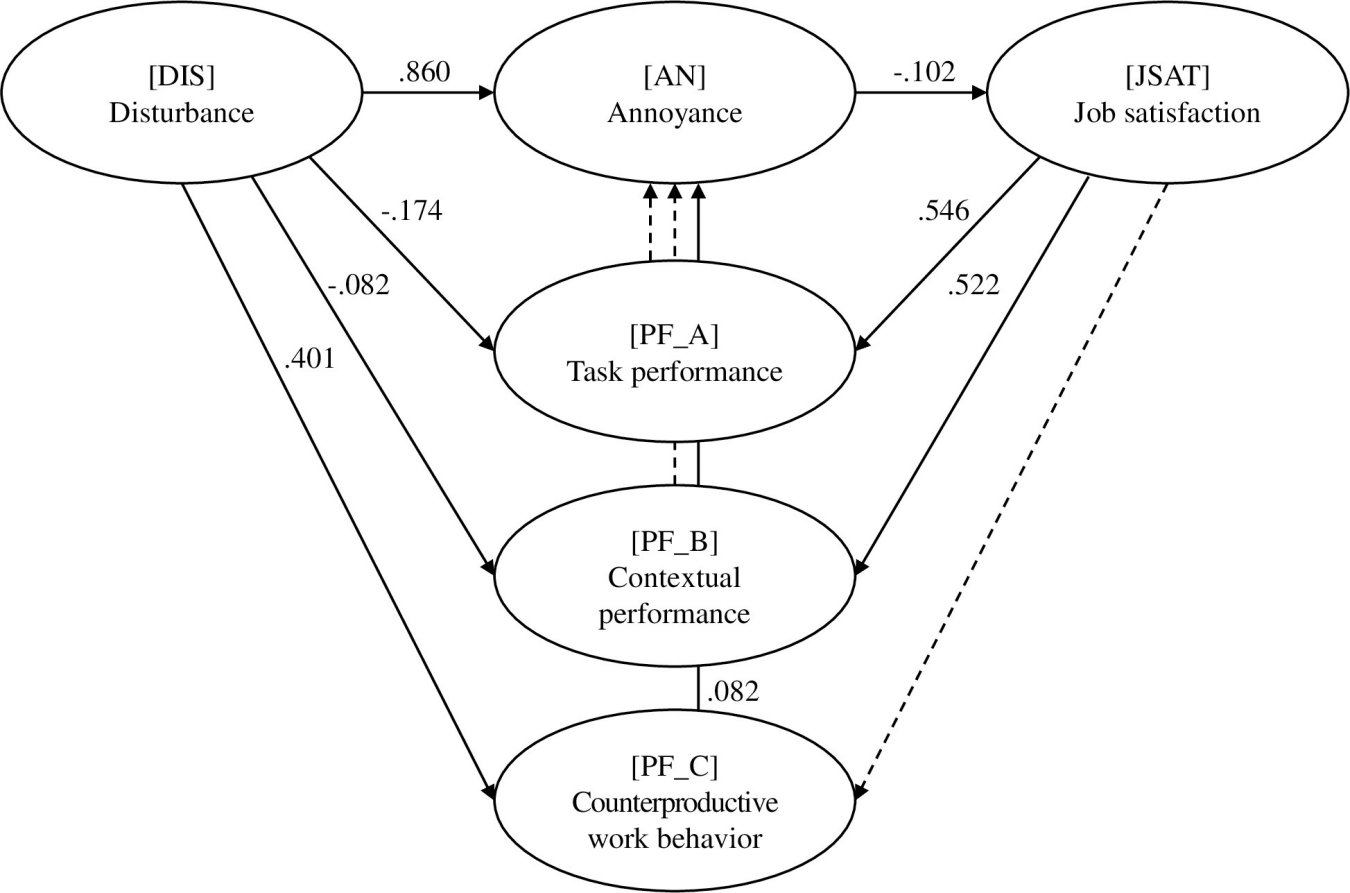
Results of the path analysis (solid line = significant; dotted line = not significant).

Path analysis was performed to test the hypothesized model. Table 5 and Fig 3 present the results of this analysis. First, the disturbance (DIS) had a significant impact on annoyance (AN), with *β* = .860 (p < .001). Second, disturbance (DIS) had negative relationships with task performance (PF_A) and contextual performance (PF_B), where *β* was–.174 (p < .001) and–.082 (p < .01), respectively, while it had a positive relationship with counterproductive work behavior (PF_C) (*β* = .401, p < .001). Third, only counterproductive work behavior (PF_C) showed a significant effect on annoyance (AN) (*β* = .082, p < .001), indicating a mediating effect of counterproductive work behavior (PF_C) between disturbance (DIS) and annoyance (AN). Fourth, there was a significant negative relationship between annoyance (AN) and job satisfaction (JSAT), with *β* = −.102 (p < .01). Finally, job satisfaction (JSAT) significantly affected task performance (PF_A) and contextual performance (PF_B), where β was .546 (p < .001) and .522 (p < .05), respectively. As Table 4 shows, the model fit indices indicated that the model had a good fit.

**Table 5:** Results of the path analysis (* p < .05; ** p < .01; *** p < .001).

*Hypothesis No*.	*Tested path*			*β*	*p*
H1	Disturbance (DIS)	→	Annoyance (AN)	.860	***
H2	Disturbance (DIS)	→	Task performance (PF_A)	–.174	***
	Disturbance (DIS)	→	Contextual performance (PF_B)	–.082	**
	Disturbance (DIS)	→	Counterproductive work behavior (PF_C)	.401	***
H3	Task performance (PF_A)	→	Annoyance (AN)	.013	
	Contextual performance (PF_B)	→	Annoyance (AN)	.002	
	Counterproductive work behavior (PF_C)	→	Annoyance (AN)	.082	***
H4	Annoyance (AN)	→	Job satisfaction (JSAT)	–.102	**
H5	Job satisfaction (JSAT)	→	Task performance (PF_A)	.546	***
	Job satisfaction (JSAT)	→	Contextual performance (PF_B)	.522	*
	Job satisfaction (JSAT)	→	Counterproductive work behavior (PF_C)	–.008	

Model fit indices: χ^2^/df = 2.831; GFI = .944; CFI = .979; TLI = .974; RMSEA = .041 (90% CI: .038; .044)

As shown in Fig 3, PF_C was a mediating variable between DIS and AN. The bias-corrected bootstrap confidence intervals calculations (1,000 bootstrap samples with 95% confidence intervals) revealed the standardized total, direct, and indirect effects of DIS on AN to be significant, with *β* = .890, .860, and .030, respectively. Additionally, results of the Sobel test [50, 51] showed that the mediation effect of PF_C between DIS and AN was significant (*z* = 3.53, p < .001).

## 4 Discussion

The study results have identified notable implications. It was found that increased indoor noise disturbance during the pandemic period led to higher levels of noise annoyance, which is consistent with the findings of previous studies [16–20]. This suggests that the degree of noise disruption is closely linked to individuals’ perception of noise annoyance, which in turn can impact their job satisfaction. Moreover, previous research showed that performance of individuals is affected by different acoustic conditions [52]. A study comparing sound insulation conditions revealed that poorer sound insulation led to worse cognitive performance [53]. The present study confirmed that noise disturbance has a significant effect on work performance. Specifically, the results suggested that noise disturbance negatively impacts both task and contextual performance. Task performance and contextual performance refer to an individual’s defined role in achieving organizational objectives, while contextual performance relates to behaviors that contribute to the social and psychological environment of the organization [54–56]. Thus, the results implied that noise disturbance has adverse impacts on essential dimensions for achieving an organization’s goals. The study also found that counterproductive work behavior increases as noise disturbance increases, with negative implications for organizations. Furthermore, the present study revealed that job satisfaction was found to have a significant influence on task performance and contextual performance, emphasizing the need for improving job satisfaction as it strengthens these two performance dimensions that contribute to the achievement of organizational goals [54–56]. However, the study also found that job satisfaction did not have a significant impact on counterproductive work behavior, indicating that individuals’ job satisfaction does not affect their tendency to engage in voluntary harmful behaviors towards their organization [54]. The path analysis showed that task performance and contextual performance did not significantly impact annoyance, which may indicate that none of these work performance attributes mediate the relationship between job satisfaction and annoyance. Conversely, the study revealed that annoyance played a mediating role in the relationship between counterproductive work behavior and job satisfaction. Higher levels of counterproductive work behavior led to increased annoyance, which in turn adversely affected job satisfaction. Besides, the investigation of the impact of work performance on annoyance was based on the stress theory [34]. From the results, it was found that counterproductive work behavior affected by negative noise perception can have an after-effect on an individual’s noise perception when exposed to indoor noise again.

The study results may raise questions to be answered; also, limitations in the survey methods should be discussed. In regression analysis, multicollinearity is strictly examined, while in structural equation modeling, it is acknowledged that independent factors may be highly correlated to some extent. Two methods have been proposed to address multicollinearity in structural equation modeling, namely variable removal and variable integration [57, 58]. Unfortunately, the method of removing variables suspected of multicollinearity is often used as a temporary solution, which can lead to model specification errors or distort the results. Another approach is to integrate highly correlated variables suspected of multicollinearity into one variable. However, this method may not provide useful information on the main research variables, make it difficult to compare with previous studies, and fail to support the newly created concepts theoretically. In the present study, the relationship between disturbance and annoyance may fall under such case. However, these two variables were neither integrated nor removed since such an approach can lead to the loss of important information and make it difficult to compare our results with previous studies. Instead, the study evaluated multicollinearity by analyzing discriminant validity proposed by Fornell and Larcker, with the results presented in Table 4. In addition, it is worth noting that while the present study recruited participants who had experience working from home during the pandemic, the survey did not limit participants to those working from home at the time of the survey. It is possible that some participants might have already returned to the office, which could have affected their ability to recall their perceptions of working from home. However, it should be emphasized that the survey was conducted in September and October of 2022, a period during which South Korea was still experiencing a high number of confirmed COVID-19 cases and a significant percentage of industries continued to implement remote work policies. Given these circumstances, it is reasonable to assume that the majority of participants were still working from home at the time of responding to the survey, or at the very least, would have been able to clearly recall their perceptions of working from home. Thus, it is important to take into account the context and timing of the study when interpreting the results, and to consider the potential impact of any limitations on the findings.

The study identified areas for future research. Since the study identified the residents living in the same dwelling as the primary source of indoor noise, highlighting the need for further investigation in future studies. The WHO recognizes noise annoyance as an adverse health effect [59, 60]. The latest document contains several systematic reviews on the diverse health effects of different noise sources [60]. In this new era after the COVID-19 outbreak, there is a need for researchers to expand their research frames to cover indoor noise, which is an emerging noise source. Another important area of future research could focus on the potential impact of various factors on residents’ noise perception. While noise has been found to be a significant factor affecting remote work environments, it is essential to consider the influence of other factors, such as visual distractions, temperature, or air quality, on individuals’ overall perception of the remote work environment. Moreover, auditory sense has been acknowledged to correlate with other senses individuals’ perception [61–63]. Future studies could also explore cross-modality of individuals’ senses and perception to gain a more comprehensive understanding of the impact of various factors on remote work environments. This may provide insights into effective strategies for improving work efficiency and job satisfaction in the context of remote work. In addition, despite the critical impact of the acoustic environment on performance [64], studies on noise-controlling strategies and room acoustic design in remote workplaces are limited. The findings in this study suggest that practical solutions for noise control and acoustic design are necessary to support employees’ performance while working remotely.

## 5 Conclusions

This study explored the effectiveness of remote work during the COVID-19 pandemic from the perspective of indoor noise perception. Analyzing the path model, the study examined the relationships between disturbance, noise annoyance, work performance, and job satisfaction perceived during the pandemic. The findings highlighted that indoor noise of occupants in the same house was a significant noise source, and this type of noise source should be further investigated in the new normal state. With remote work likely to remain a part of our lives, the study’s implications become all the more crucial. The study emphasized that indoor noise should be considered as a significant factor affecting work performance and job satisfaction in remote work environments. Since the study solely relied on self-reported data, it might have been subject to recall bias. Future research should provide a more accurate assessment of indoor noise levels by collecting objective noise measurements along with the self-reported data. In addition, future research should aim to investigate the effectiveness of different acoustic design solutions for remote work environments. Another area of research could focus on the impact of noise on specific job types, such as creative or analytical work. Practically, the study’s findings have important implications for designing homes with acoustics in mind. With more people working from home, homes are becoming multi-functional spaces that require effective acoustic designs to ensure a productive and conducive environment for various purposes.

## Supporting information

S1 Data (XLSX)
